# Quality of Vermicompost and Microbial Community Diversity Affected by the Contrasting Temperature during Vermicomposting of Dewatered Sludge

**DOI:** 10.3390/ijerph17051748

**Published:** 2020-03-07

**Authors:** Hongwei Zhang, Jianhui Li, Yingying Zhang, Kui Huang

**Affiliations:** School of Environmental and Municipal Engineering, Lanzhou Jiaotong University, Lanzhou 730070, China; zhw@mail.lzjtu.cn (H.Z.);

**Keywords:** earthworms, microorganisms, sludge recycling, temperature, vermicomposting

## Abstract

This study aimed to investigate the effects of temperature on the quality of vermicompost and microbial profiles of dewatered sludge during vermicomposting. To do this, fresh sludge was separately vermicomposted with the earthworm *Eisenia fetida* under different temperature regimes, specifically, 15 °C, 20 °C, and 25 °C. The results showed that the growth rate of earthworms increased with temperature. Moreover, the lowest organic matter content along with the highest electrical conductivity, ammonia, and nitrate content in sludge were recorded for 25 °C indicating that increasing temperature significantly accelerated decomposition, mineralization, and nitrification. In addition, higher temperature significantly enhanced microbial activity in the first 30 days of vermicomposting, also exhibiting the fastest stabilization at 25 °C. High throughput sequencing results further revealed that the alpha diversity of the bacterial community was enhanced with increasing temperature resulting in distinct bacterial genera in each vermicompost. This study suggests that quality of vermicompost and dominant bacterial community are strongly influenced by the contrasting temperature during vermicomposting of sludge, with the optimal performance at 25 °C.

## 1. Introduction

With the development of China in recent years, the government has also built thousands of wastewater treatment plants, generating large amounts of dewatered sludge with several pollutants that are difficult to be treated [1,2]. Accordingly, the total amount of dewatered sludge in China is expected to exceed 50 million tons (80% water content) in 2020 [3]. Compared with incineration and landfills, the sludge fertilizer product combined with the agricultural use is deemed as a potential method of recycling to resolve the sludge problem in China [1] based on the control standards for sludge products for agricultural use (GB 4284-2018). Vermicomposting is a biochemical method for converting sludge into high-value organic microbial fertilizer by the joint action of earthworms and microorganisms [4,5,6,7]. As a green technology, plenty of vermicomposting factories for treating sludge have been recently established in China allowing them to profit highly from vermicompost and vermiculture in the process.

In a vermicomposting system, both earthworms and microorganisms play important roles in the decomposition and conversion of sludge [8]. Relative to microbes, earthworms make a larger contribution to sludge stabilization through gut digestion, mucus production, and then casting. This makes earthworms significant in vermicomposting. Accordingly, many environmental factors, such as temperature, moisture, noise, and light, may also influence the growth of earthworms, and thus, also modify properties of the final products [4,5]. Among these variables, temperature is considered one of the most critical factors affecting the growth and reproduction of earthworms [9,10]. For instance, *Eisenia fetida* as a ubiquitous epigenetic species capable of vermicomposting exhibited wide tolerance to a broad temperature range (15–25 °C) for their growth [11]. Previous studies also indicate high variability in the temperature used for vermicomposting with most using room temperature, also suggesting a wide range of temperature useful for treating sludge [6,12,13]. Only a few publications clearly reported that 20 °C was the optimal temperature condition for treating activated sludge [14]. Given that the growth of earthworms is strongly associated with temperature, different regimes may also affect the quality of the vermicomposting product and its agricultural value. Hence, understanding the optimal temperature condition to operate vermicomposting of sludge is vital to drive the development of the vermicomposting industry.

Although the use of earthworms is popular in vermicomposting systems, microorganisms also play important roles in decomposing and converting the organic matter present in sludge [8,15]. In addition, the dewatered sludge is mainly characterized by active microbes and debris from dead cells and particulate matter [1]. Consequently, the activity, abundance, and community composition of microbes in sludge may be strongly associated with the vermicomposting efficiency and sludge quality [8,13]. For other sludge biodegradation systems, the impact of temperature on microbial profiles has been well-investigated, such as on anaerobic digestion [16,17], composting [18,19], and vermifiltration [20]. Even in soil systems, temperature also showed an intensive effect on microbial growth, activity, and community structuring within a short period, and thus affects decomposition of organic substances [21,22,23]. However, until now, the effect of temperature on microbial activity, abundance, and community composition during vermicomposting of sludge remains unclear, while it is strongly associated with the quality of vermicompost.

The objectives of this study then were to investigate the effect of temperature on vermicomposting quality for treating sludge, and to understand the effect of temperature on microbial activity, abundance, and community structuring during vermicomposting. Taking into account that the optimal temperature for the growth of *Eisenia fetida* was from 15 to 25 °C, three vermicomposting treatments for recycling sludge at 15 °C, 20 °C, and 25 °C were compared in this study.

## 2. Materials and Methods

### 2.1. Experimental Setup

The earthworm *Eisenia fetida*, which had been cultured by dewatered sludge for 2 years in the laboratory, was selected for the vermicomposting experiment. The dewatered sludge was obtained from Lanzhou Qilihe wastewater treatment plant, Anning District, Lanzhou, China. Based on the previously used method [12], the dewatered sludge was pelleted into small particles of 5 mm size to enhance oxygenation of the sludge. The main properties of the sludge used are summarized in Table 1.

Since 15–25°C had been reported to be suitable for the growth of *Eisenia fetida* [24], three temperature regimes including 15, 20, and 25 °C were separately established for the vermicomposting of sludge. Nine [11] metal pots with the size of 36 cm × 12 cm (diameter × height) were used as vermireactors, with triplicate for each treatment. For each reactor, 100 young and active earthworms with an average weight of 0.5 g were inoculated into each vermireactor filled with 4 kg fresh sludge. This density of earthworms was based on the previous studies [6,12]. To maintain the moisture of 70–80%, each reactor was covered with a plastic film with some small holes. Subsequently, three replicate reactors were placed into three incubators (Yiheng, Shanghai, China) with constant temperatures of 15 °C, 20 °C, and 25 °C, respectively. During the experiment, all reactors were turned over twice a week to homogenize the vermicompost. After 60 days, the experiments were stopped, and the earthworms and their cocoons were weighed and counted separately. The samples were collected at the interval of 10 days, and subsequently stored in 4 °C and −20 °C for further use.

### 2.2. Physicochemical and Enzymatic Properties

The water and organic matter content were determined by drying the samples in the oven for 12 h at 105 °C and for 6 h at 550 °C, respectively. The mixture of dry sample and distilled water (1:50, w/v) was used to measure the pH and electrical conductivity using a pHS-3C acidometer and a conductivity meter, respectively. The same water mixture was used to determine the ammonia-nitrogen (NH_4_^+^), nitrate-nitrogen (NO_3_^−^), and available phosphate (PO_4_^3−^) with the spectrophotometric methods. The fresh sludge was fumigated with chloroform before being used to measure the microbial biomass carbon (MBC) via the spectrophotometric method following Fu et al. [12]. Dehydrogenase activity (DHA) was also determined to evaluate microbial activity using the triphenyl tetrazolium chloride (TTC) method [6].

### 2.3. DNA Extraction, PCR and Sequencing

The commercially available Power Soil^®^ DNA Isolation Kit (MO BIO, Carlsbad, CA, USA) was used for DNA extraction without modification. Before being stored at −40 °C, the extracted DNA was diluted 20-fold using DNA-free water to decrease the pollutants.

V3-V4 regions of the bacterial 16S rRNA gene were amplified using the universal primers of 304f-806r [6]. The Polymerase chain reaction (PCR) was carried out in a 25 µl mixture comprised of 1 μL DNA template, 2.5 μL of each forward and reverse primer (0.5 μM each), 25 μL polymerase (Phusion^®^ High-Fidelity PCR Master Mix, New England Biolabs, Beijing, China), and 19 μL DNA-free water. The amplification conditions with 30 cycles included denaturation at 94 °C for 30 s, annealing at 58 °C for 1 min, and extension at 72 °C for 1 min. After checking by electrophoresis with 2% agarose gel, the PCR products were recovered and then purified with a GeneJET Gel Extraction Kit (Thermo Scientific, Shanghai, China). The sequence library was built with an Ion Plus Fragment Library Kit 48 rxns (Thermo Fisher, Shanghai, China) and then quality checked with a Qubit^®^ 2.0 Fluorometer (Thermo Scientific, Shanghai, China). The sequencing was performed on a Life Ion S5^TM^ platform at Novogene Bioinformatics Technology Co., Ltd. (Beijing, China). After removing the low-quality parts of the sequences with the Cutadapt software (V1.9.1, Stockholm, Sweden), the reads were compared with the UCHIME algorithm (V11, Tiburon, CA, USA) and the Gold database of genomes (V7, Los Angeles, CA, USA). Quality reads were then clustered to generate operational taxonomic units (OTUs) at the 97% similarity level using the UPARSE package (V7.0.1001, Los Angeles, CA, USA). A representative sequence of each OTU was assigned taxonomy in the Ribosomal Database Project (RDP) classifier (V11, East Lansing, MI, USA) [6].

### 2.4. Statistical Methods

One-way ANOVA was used to evaluate differences between treatments with a significant level at *p* < 0.05 using the SPSS 17.0 software. Such parameters as organic matter (OM), dissolved organic carbon (DOC), electrical conductivity (EC), NH_4_^+^/NO_3_^−^, microbial biomass carbon (MBC), and dehydrogenase activity (DHA) were used to evaluate significant correlated factors affecting stabilization of vermicomposting under three temperature conditions, which were plotted using principal component analysis (PCA) implemented in the Statistica 10.0 software (Statsoft Inc. Tulsa, USA). A heatmap diagram of the dominant bacterial community was drawn using the Heml 1.0 software (Wuhan, China). The Venn diagram showing the extent of overlap in bacterial OTUs (at the 3% evolutionary distance) among the different treatments was done in the Origin 8.0 software (OriginLab, Northampton, MA, USA).

## 3. Results

### 3.1. Changes in Physicochemical Properties

After the experiment, the observed growth rates of earthworms at 15 °C, 20 °C, and 25 °C were 0.43, 0.82, and 1.17 mg/worms/day, respectively. Those at 25 °C showed 90.7% and 172% higher growth rates than at 20 °C and 15 °C, respectively. These results clearly indicate that higher temperature also stimulated increased growth rates of earthworms consistent with previous observations [25,26]. However, Hait and Tare [14] reported that 20 °C was the optimal temperature condition for treating activated sludge, which could be due to the differences in the substrate used in vermicomposting.

**Figure 1:**
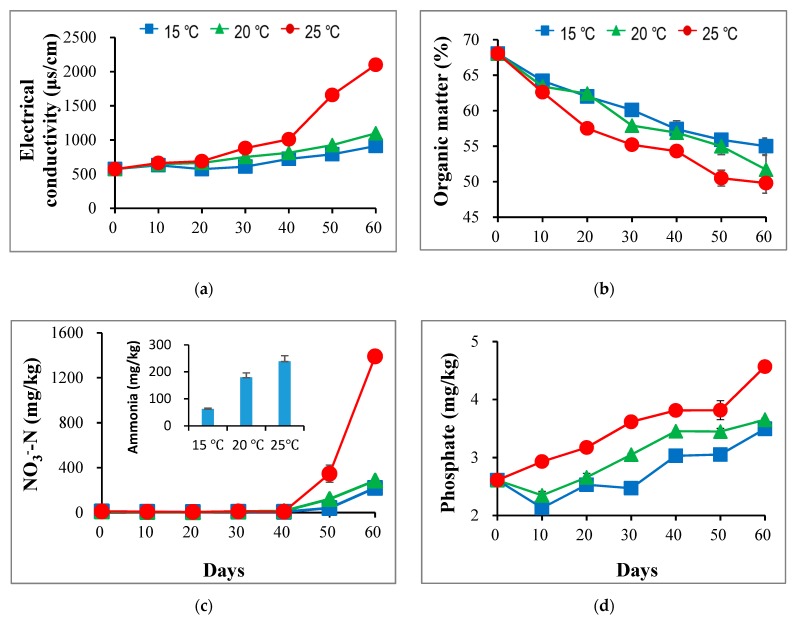
Changes of electrical conductivity (**a**), organic matter (**b**), ammonium, nitrate (**c**), and phosphate (**d**) levels during vermicomposting of sludge at 15 °C, 20 °C, and 25 °C, respectively.

During vermicomposting, pH values showed little fluctuation, which ranged from 6.5 to 7.1 in the three treatments. Towards the end of the incubation, no significant difference in pH was observed, indicating that the effect of temperature on the pH value was insignificant. In contrast, electrical conductivity displayed a gradual increment towards the end of the experiment (Figure 1a). The increasing electrical conductivity could be due to the remineralization of organic substances in dewatered sludge [14]. For the final vermicomposting product, the electrical conductivity at 25 °C was significantly higher than at 20 °C and 15 °C (1.91 and 2.30 times, respectively). Such findings suggest that higher temperature could strongly accelerate remineralization in sludge during vermicomposting.

As shown in Figure 1b, the organic matter in all treatments markedly decreased during vermicomposting, showing the largest drop at 25 °C. In the end, the OM dropped by 19.1%, 25%, and 26.7% in the reactors at 15 °C, 20 °C, and 25 °C, respectively. This shows that conditions with higher temperature also had a higher decomposition rate of organic matter in the sludge. Similarly, the highest removal rate of volatile suspended solids was found in the seasons with higher temperature in the vermifilters for treating excess sludge [20]. However, Garg and Gupta [26] argued that OM degradation rate of household waste was higher in winter than that in the summer season in India during the vermicomposting process. Combined with the earlier finding of the earthworms’ growth rate in this study, it may be deduced that higher temperature could have first stimulated the earthworms’ activity before promoting the decomposition of OM.

Dewatered sludge is mainly comprised of microorganisms and their metabolic products, where proteins tend to have a larger share than other compounds, such as saccharides, lipids, and nucleic acid [1]. Therefore, degradation and transformation of nitrogenous substances were closely associated with stabilization of the sludge. Generally, NH_4_^+^ and NO_3_^−^ are considered important indices for assessing the vermicomposting stabilization process [27,28]. As shown in Figure 1c, the highest ammonification rate was recorded in the 25 °C reactor, significantly higher than for the other two treatments. Accordingly, the dewatered sludge is mainly comprised of proteins, microorganisms, and some organic substances [13]. The higher ammonification rate in the 25 °C reactor may be linked with the organic matter decomposition, especially of protein-like substances. Meanwhile, the NO_3_^−^ content in all treatments was close to zero until after 40 days, which was followed by a drastic increase towards the end of the experiment (Figure 1c). The sharp increase in the nitrate level from the mid-phase of vermicomposting is consistent with previous reports [6,12]. Finally, the largest increase was observed at 25 °C, followed by the treatments of 20 °C and 15 °C, indicating that higher temperature can also induce nitrification. This could be because ammonia-oxidizing microorganisms are more active at 25 °C than at 15 °C and 20 °C [29]. Gubry-Rangin et al. [30] also reported that the optimum temperature for nitrification is 20 °C to 30 °C, depending on the pH value of the surrounding environment.

In addition, orthophosphate levels exhibited a continuous enhancement in all treatments for the duration of the entire experiment as illustrated in Figure 1d. Like other inorganic salts, the highest orthophosphate level was also observed at 25 °C. Generally, accumulation of orthophosphates in the sludge vermicompost may probably be related to sludge remineralization facilitated by earthworms, corroborating previous reports [6,12]. As a result, the highest electrical conductivity observed at 25 °C was consistent with the highest concentration of orthophosphates observed in the present study.

### 3.2. Changes in Microbial Activity and Abundance

**Figure 2:**
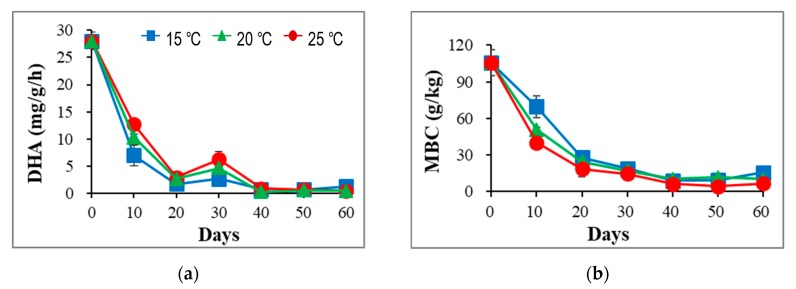
(**a**) Changes of dehydrogenase activity (DHA) and (**b**) microbial biomass carbon (MBC), during vermicomposting of sludge at 15 °C, 20 °C, and 25 °C, respectively.

The results showed (Figure 2) that both microbial activity and biomass in the sludge showed rapid reduction in all treatments during the incubation, which was also observed in previous studies of vermicomposting for dewatered sludge [12,13].

Such findings may be due to the rapid loss of high OM and MBC in the initial sludge. For the DHA, the higher activity was observed at 25 °C during the first 30 days, which was earlier compared to the other two treatments. These findings suggest that temperature can enrich microbial activity during the first phase of vermicomposting, which may also imply that sludge decomposition mainly occurred in the first 30 days. As a result, no significant difference in the DHA was observed among the three treatments after 30 days, which may be ascribed to the loss of available OM for microbial growth. The temperature fluctuation positively correlated with microbial activity, which has also been detected in several biosolid treatment systems [17,18,20].

As for the MBC, temperature showed negative effects on microbial biomass (Figure 2), opposite to what has been reported by other studies [23,31]. Apparently, it seems that higher temperature decreased the abundance of the active microbial population during the vermicomposting of sludge. In fact, the MBC was strongly linked with the OM concentration, which is an intermediate product of decomposition that can be rapidly transformed and decomposed by microbial activity [23]. Hence, the larger loss of the MBC at 25 °C could probably be due to faster transformation and decomposition of OM during vermicomposting. Moreover, higher temperature may directly enhance the activity of earthworms, boosting the predatory effects of earthworms on the microbial population. In addition, a related study documented that temperature had only minor effects on both bacterial and fungal growth below 30 °C [32].

### 3.3. Stabilization Process. of Vermicomposting

**Figure 3:**
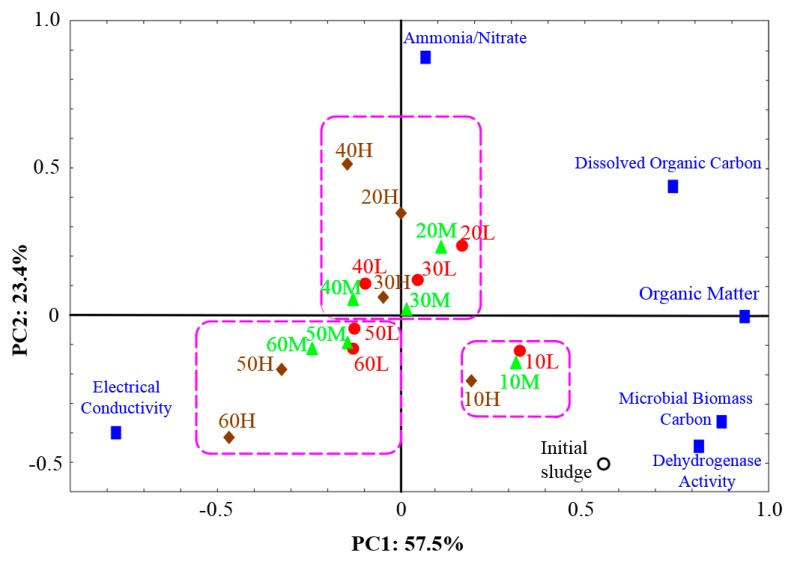
Principal component analysis of vermicomposting of sludge under three different temperature conditions. Letters “L”, “M”, and “H” represent the temperatures of 15 °C, 20 °C and 25 °C, respectively. The numbers at the head of L, M, and S mean vermicomposting samples collected on day 10, 20, 30, 40, 50, and 60. The blue diamond blocks mean the different parameters of stability. The green dotted cycles represent the different stages of vermicomposting.

As depicted in Figure 3, the cumulative variance of 80.9% and high loading rate of all parameters (more than 0.75) in the first and the second principal components indicate that the selected parameters were reasonable to be used in assessing the stabilization process in the present study. Specifically, OM, DOC, MBC, and DHA were significantly positively correlated with the first principal component, which indicates capability for decomposition and remineralization of sludge. In addition, NH_4_^+^/NO_3_^−^ had a significant positive correlation (*r*^2^ = 0.92) with the second principal component, possibly reflecting ammonification and ammonia oxidation processes.

Based on Figure 3, the vermicomposting process could be divided into three stages: days 0–10, days 10–40, and days 50–60. Specifically, in the first stage, the temperature began to affect the decomposition, since the projective position to the 25 °C treatment significantly differed with the other two treatments in the fourth quadrant. In the second stage, all points were distributed in the first and the second quadrants with a positive relationship with NH_4_^+^/NO_3_^−^ suggesting that intense ammonification occurred during this stage, especially in the 25 °C reactor on day 40. In the last stage, all treatments positioned in the third quadrant also positively correlated with EC, but negatively with OM, DOC, MBC, DHA, and NH_4_^+^/NO_3_^−^. During the entire process, the 25 °C treatment significantly differed with the other two treatments implying that the sludge could be rapidly stabilized at 25 °C. Consequently, it can be inferred that higher temperature could have positive effects on stabilization of vermicomposting. Further, it does mean that the sludge vermicompost obtained at 25 °C is better as a soil fertilizer, as suggested by [15].

### 3.4. Changes in Microbial Community Structure

After sequencing, a total of 61,873, 65,436, and 66,713 quality reads were generated from the 15 °C, 20 °C, and 25 °C treatments, respectively. As shown in Table 2, the Shannon and Chao1 indices had the highest values at 25 °C, followed by the 15 °C and 20 °C treatments. These results indicate that both community diversity and richness were promoted by higher temperature during vermicomposting. This result coincided with that of Wu et al. [21], who reported that the alpha diversity of soil gradually increased along a temperature gradient from 10 to 30 °C, but sharply decreased at 40 °C.

**Figure 4:**
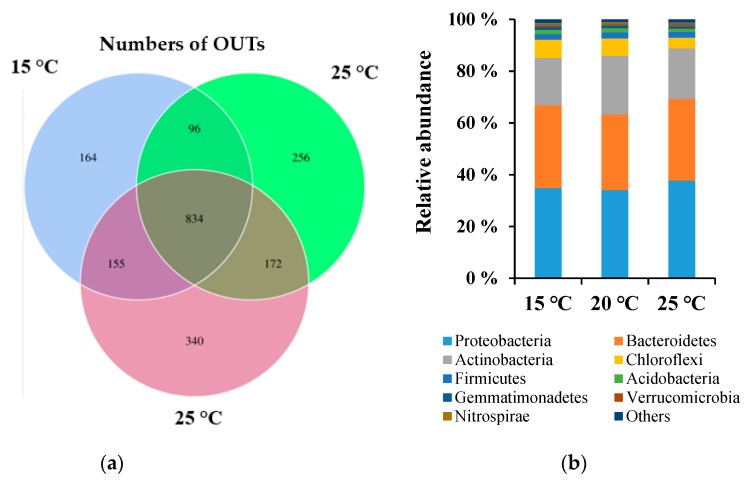
Bacterial components of operational taxonomic units (OTUs) number (**a**) and phylum communities (**b**) at the different treatments.

In contrast, the Simpson index showed the values for the three treatments were even higher than 0.97, and no obvious difference was recorded between these treatments, suggesting that temperature did not affect microbial evenness. Further, the observed amount of bacteria (Figure 4a) at 15 °C, 20 °C, 25 °C was 1249, 1358, and 1501, respectively. The amount of bacteria at 15 °C was lower by 8.7% and 20.2% than at 20 °C and 25 °C, respectively. In addition, the specific species at 15 °C, 20 °C, and 25 °C amounted to 164, 256, and 340, respectively. These results suggest that high temperature conditions can strongly increase the number of bacterial species, forming a different bacterial community in the final vermicompost.

As summarized in Figure 4b, the classified OTUs at the phylum level showed a shift in the bacterial community structure between the three temperatures. The 15 °C treatment was dominated by the Proteobacteria (34.1%), Bacteroidetes (32.1%), and Actinobacteria (18.2%). In comparison, Proteobacteria (34.8%) also dominated at 20 °C, followed by Bacteroidetes (30.2%) and Actinobacteria (19.2%), as well as at 25 °C (Proteobacteria 37.7%, Bacteroidetes 31.6%, Actinobacteria 20.6%). These observations were consistent with previous reports that the same group dominated in the sludge vermicompost [6,27]. In addition, Proteobacteria and Actinobacteria showed relative sensitivity to temperature, since their shares increased with temperature. The positive relationship between temperature and the shares of Proteobacteria and Actinobacteria was also observed previously [21].

**Figure 5:**
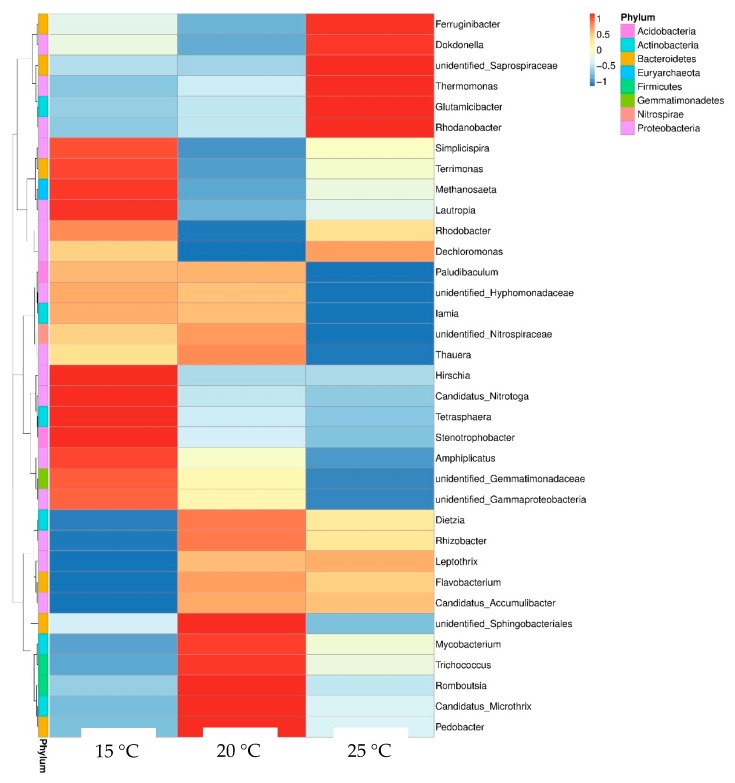
Heat map diagram of dominant bacteria for different sludge treatments at the genus level.

Notably, Chloroflexi displayed an opposite trend to the increasing temperature in this study. As exhibited in Figure 5, the dominant genera of bacteria showed a dissimilar pattern with temperature change during vermicomposting of sludge (Figure 5). The 15 °C treatment was dominated by *Terrimonas* (3.9%), *Amphiplicatus* (2.2%), *Tetrasphaera* (1.1%), and others genera, including *Methanosaeta*, *Lautropia*, *Stenotrophobacter*, and unidentified *Gemmatimonadaceae*. The *Candidatus Microthrix* (11.6%) was the predominant taxon at 20 °C, followed by *Trichococcus* (5.3%) and *Mycobacterium* (1.2%). Additionally, members of *Dokdonella* (10.7%), unidentified *Saprospiraceae* (6.5%), *Rhodanobacter* (2.1%), and *Thermomonas* (1.6%) were the dominants at 25 °C. Such results indicate that temperature can strongly influence bacterial community structuring in the final sludge vermicompost. These results were consistent with previous studies of soil [21], compost [19], and anaerobic systems [17]. Higher temperature may enhance mortality and reduce the stochastic processes of birth and colonization of microorganisms [22], thus promoting diversification in ecological niches. Wu et al. [21] demonstrated that every 1 °C elevation in soil temperature could lead to a change of about 36.5% in the community structure of bacteria per year.

In addition, the predominant bacterial taxon is strongly associated with the vermicomposting function. For instance, the presence of *Terrimonas*, a member of the family Chitinophagaceae, could indicated high degradation, since members of this genus have been previously shown to actively degrade organic compounds and cellulose, which are also often isolated from the activated sludge [13,33]. *Candidatus Microthrix*, on the other hand, are ubiquitous lipid-accumulating filamentous bacteria that not only cause bulking and foaming, but can also assimilate diverse carbon substrates while being adaptable to a wide range of environmental conditions [34]. *Dokdonella*, which are strictly aerobic and urease-negative rods with an optimum growth temperature of 40 °C, are also usually found in compost [35] and vermicompost [13]. Hence, in this study, the striking difference in the composition of bacterial genera in contrasting environments may directly affect stabilization of the sludge. Although 30 °C may be an optimal temperature for bacterial growth and community succession, *E. fetida* was not able to survive in the warmer condition, affecting the vermicomposting process. Thus, it seems that 25 °C could be the best vermicomposting condition, as it exhibited the highest activities of both earthworms and microorganisms, making it an optimal temperature for vermicomposting of dewatered sludge. On the other hand, since the highest temperature is only 25 °C, the sanitization for some biological pollutants, such as pathogens, antibiotic resistance genes, and viruses, is not complete in sludge vermicompost, because they are generally inactivated by processes involving increase in temperature [36]. Similarly, the heavy metals may not be effectively removed at this temperature. Therefore, the final environmental risk of sludge vermicompost should be assessed before it is used as a sludge fertilizer. Furthermore, the effects of temperature on reduction in toxicants in sludge during vermicomposting require further research.

## 4. Conclusions

The results obtained from this study indicate that temperature significantly affected decomposition and mineralization of sludge during vermicomposting, with the fastest stabilization process occurring at 25 °C. Higher temperature enhanced both microbial activity and earthworms’ growth in the first 30 days, which could have rapidly stabilized the sludge. In addition, the alpha diversity of the bacterial community was strongly stimulated by a higher temperature, showing a distinct dominant genus in the final vermicompost. This study suggests that 25 °C is the optimal temperature for vermicomposting of dewatered sludge with *E. fetida*. However, the environmental risk of heavy metals and pathogens in sludge vermicompost should be assessed before it is used as a sludge fertilizer.

## Figures and Tables

**Table 1:** Physicochemical properties of the initial sludge and final vermicomposting products at 15 °C, 20 °C, and 25 °C. Value are means ± SE (*n* = 3).

Parameters	Initial Sludge	Vermicomposting after 60 days		
		15 °C	20 °C	25 °C
Water content (%)	80.38 ± 0.01	80.15 ± 0.0	80.37 ± 0.0	80.53 ± 0.0
Organic matter (%)	68.0 ± 7.7	55.0 ± 1.16	51.7 ± 1.99	49.8 ± 1.42
pH	6.77 ± 0.005	6.53 ± 0.005	6.77 ± 0.00	7.01 ± 0.00
Electrical conductivity (μs/cm)	573 ± 8.49	911.5 ± 2.04	1097.5 ± 6.94	2100 ± 16.33
Dissolved organic carbon (mg/kg)	16.69 ± 0.13	14.05 ± 0.04	13.92 ± 0.04	9.17 ± 0.05
Ammonium (mg/kg)	7.36 ± 0.08	103.79 ± 0.76	206.06 ± 0.23	238.91 ± 2.9
Nitrate (mg/kg)	10.26 ± 2.0	219.91 ± 45.15	285.10 ± 9.10	1389.49 ± 47.07
Ammonium/nitrate	0.74 ± 0.19	0.24 ± 0.04	0.67 ± 0.04	0.13 ± 0.05
Microbial biomass carbon (g/kg)	105.61 ± 10.6	15.63 ± 0.98	8.50 ± 0.18	6.71 ± 1.08
Dehydrogenase activity (mg/g/h)	28.06 ± 1.34	0.49 ± 0.08	0.49 ± 0.02	0.27 ± 0.01

**Table 2:** Alpha diversity of the bacterial community in sludge vermicomposting products at 15 °C, 20 °C, and 25 °C.

Sample Name	Shannon	Simpson	Chao1
15 °C	6.88	0.973	1373.25
20 °C	6.98	0.972	1508.01
25 °C	7.06	0.974	1651.31
